# Systematic review: oral and maxillofacial radiology as fundamental methods of virtual autopsy

**DOI:** 10.1093/fsr/owad028

**Published:** 2023-11-10

**Authors:** Wilma N Azizah, Fahmi Oscandar, Merry A Damayanti

**Affiliations:** Faculty of Dentistry, Universitas Padjadjaran, Bandung, Indonesia; Department of Oral and Maxillofacial Radiology-Forensic Odontology, Faculty of Dentistry, Universitas Padjadjaran, Bandung, Indonesia; Department of Oral and Maxillofacial Radiology-Forensic Odontology, Faculty of Dentistry, Universitas Padjadjaran, Bandung, Indonesia

**Keywords:** systematic review, oral and maxillofacial radiographs, virtual autopsy, forensic odontology, dental autopsy, CT scan, CBCT, MRI

## Abstract

Refusal of autopsy occurs for various reasons, including religious beliefs and the risk of infectious diseases such as coronavirus disease 2019. Dental autopsy involves invasive procedures, including incision and access openings in the oral cavity. Radiographic techniques can be used as an alternative to the conventional autopsy process in the field of forensic odontology, providing a non-invasive approach that does not involve tissue damage. The current study aimed to analyse the current status of the applicability of oral and maxillofacial radiology for virtual forensic odontological autopsy. A systematic review was conducted in accord with the Preferred Reporting Items for Systematic Review and Meta-Analysis protocol. The literature search was conducted from December 2021 to October 2022 using the PubMed, ScienceDirect, and SAGE Journals databases. Article selection was carried out by eliminating duplication, screening titles and abstracts, and reading the entire content of the article. A thematic analysis method was used to identify themes in the collected data. A total of 15 articles were included, and several uses of oral and maxillofacial radiography in virtual autopsy and forensic odontology were identified. Four techniques were identified that can be used for virtual autopsy in forensic odontology. The use of computed tomography (CT) scanning for virtual autopsy was reported in six articles, cone beam CT was reported in five articles, magnetic resonance imaging was reported in two articles, and multidetector CT was reported in two articles. In the studies included in this review, the identified oral and maxillofacial radiograph techniques that are used as the fundamental methods of virtual autopsy in forensic odontology are CT scanning (including multidetector CT), cone beam CT, and magnetic resonance imaging. The different methods of oral and maxillofacial radiography for virtual autopsy were identified as having advantages as well as limitations in their use in forensic odontology. Most of the included studies indicated that the virtual autopsy approach cannot yet stand alone as an identification method, but provides a useful adjunct for gathering dental evidence.

**Key points:**

- Virtual autopsy is a valuable tool for non-invasive autopsies in the field of forensic odontology.
- CT scans (including MDCT), CBCT, and MRI can be used for virtual autopsy in the identification of individuals based on dental evidence.
- The use of oral and maxillofacial radiographs can shorten the time required for forensic odontology activities.
- Artefacts caused by amalgam and other metal restorations on radiographic images can raise doubts in the identification process.
- Although the use of oral and maxillofacial radiology has many advantages, it cannot yet stand alone as an identification method.

## Introduction

The main procedure in a dental autopsy involves invasive procedures, including incisions and access openings [1]. Previous studies conducted by Oluwasola et al. [2] and Blum et al. [3] reported that the potential delay to the burial process was a factor leading to autopsy refusal in some cases. Carwen et al. [4] and Sauvegrain et al. [5] reported that the presence of fear and concern about invasive procedures being performed on the remains of relatives was another reason for autopsy refusal. Atanda et al. [6] and Malik et al. [7] reported that people of various religions, beliefs, and cultures sometimes refuse the implementation of invasive conventional autopsy. Some individuals believe that autopsy will damage the corpse, and that it does not show respect for the body [7].

The International Committee of the Red Cross classifies autopsy as a procedure that includes a high-risk level of exposure to the SARS-CoV-2 virus [8]. Plenzig et al. [9] and Gabbrielli et al. [10] reported that the SARS-CoV-2 viral genome can persist in tissues for >2 weeks postmortem. It is suspected that transmission can occur during this period. Various diseases can still be transmitted when the host is deceased, including human immunodeficiency virus (HIV) and tuberculosis (TBC) [11]. A study by Nyberg et al. [12] indicated that a corpse with HIV should be considered infectious for at least 1–2 weeks postmortem. Flavin et al. [13] reported that 67% of cases of active TBC infectious disease were only detected when the corpse was autopsied. Thus, appropriate information is needed regarding alternative techniques so that autopsy activities can still be carried out safely during pandemics.

Emergencies involving chemical, biological, radiological, nuclear, and high yield explosives (CBRNE) can result in injury, illness, or loss of life. INTERPOL [14] reported that in CBRNE disasters, bodies may be contaminated or infected by dangerous or deadly agents. Bland [15] reported that CBRNE exposure can cause poisoning, infection, irradiation, and injury. On the basis of these findings, CBRNE disaster management can pose risks not only to respondents but also to the community and the surrounding environment. Therefore, in addition to requiring a good protection strategy, handling corpses/patients from CBRNE events also requires less invasive autopsy methods to protect staff and operators from the risk of hazardous exposure.

Virtual autopsy, or “virtopsy”, is a non-invasive autopsy technique that combines three-dimensional (3D) imaging methods from ultrasonography, computed tomography (CT), and magnetic resonance imaging (MRI) data [16, 17]. A study conducted by Rudisch et al. [18] indicated that the use of ultrasound on corpses can identify the conditions of condylar erosion and disc placement of the temporomandibular joint. Another study conducted by Oesterhelweg et al. [19] reported that the use of postmortem multislice or multidetector CT (MDCT) and MRI can clearly show a bolus obstructing the larynx, thus aiding the process of determining the cause of death. El-Dahab et al. [20] reported that cone beam CT (CBCT) can be used for sex determination. Jensen et al. [21] reported that the use of postmortem CT scanning increases the efficiency of autopsy and can show images of prostheses, restorations, endodontic treatments, implants, pathological conditions of the teeth, and the number of teeth, which can assist in the corpse identification process. These previous studies indicate that the use of 3D data from ultrasound, CT scanning, and MRI can reveal information about the condition of the mouth and surrounding tissues, including the condition of the temporomandibular joint, the presence of laryngeal obstruction, sex determination, presence of restoration, and aspects of the pathological condition of the corpse’s teeth. Thus, these methods can potentially be used as an alternative to conventional invasive autopsy processes.

To the best of our knowledge, no previous study has systematically reviewed studies of virtual autopsy methods using oral and maxillofacial radiology as part of forensic odontology. Thus, it may be useful to conduct further research by reviewing various literature sources regarding this matter. The current study aimed to review the current status of the applicability of oral and maxillofacial radiology for virtual forensic odontological autopsy.

This systematic review sought to answer the following research question: what are the oral and maxillofacial radiography techniques that can be used for virtual autopsy in forensic odontology. In addition, we sought to clarify the advantages and limitations of the use of virtual autopsy in forensic odontology.

## Materials and methods

### Study design

This systematic review was conducted using the Preferred Reporting Items for Systematic Review and Meta-Analyses (PRISMA) [22] checklist as a reference for completing this research (submission number: 376690). The article search was conducted independently by the authors at the Department of Oral and Maxillofacial Radiology-Forensic Odontology from December 2021 to October 2022 using the PubMed, ScienceDirect, and SAGE Journals databases with the keywords “forensic odontology”, “forensic dentistry”, “dental autopsy”, “oral autopsy”, “virtual autopsy”, “forensic imaging”, and “virtual imaging”.

### Inclusion and exclusion criteria

We included articles that discussed virtual autopsy related to oral and maxillofacial radiography and forensic odontology that were published in English as original research articles from 2011 to 2022. The exclusion criteria in this study were used to omit articles with incomplete and inaccessible text, articles in the form of case reports and case series, review articles, research articles with non-human samples, and articles that did not discuss virtual autopsy in oral and maxillofacial radiography techniques and forensic odontology.

### Article selection

Three authors independently screened articles from the PubMed, SAGE Journals, and ScienceDirect databases. Article selection was carried out by one author and supervised by two other authors. The first selection was performed by filtering by the year of publication. Duplication filtering was performed by downloading all search results from each database in Research Information Systems (RIS) format and exporting them to Mendeley software (Elsevier, London, UK). Screening of titles and abstracts was carried out to determine the relevance of articles from the entire database. The remaining articles underwent final screening by reading the full text of the article to determine the suitability of the content in relation to the title of the study. The PRISMA [22] flow chart was used to describe the search results, selected studies, included and excluded studies, as well as the reasons for the exclusion of studies. We identified 40 irrelevant articles. Information regarding publication details (author, year of publication, journal reputation) and article details was obtained. Data were managed in Excel (Microsoft, Redmond, WA, USA).

### Data mapping and analysis

Data mapping was carried out by examining the specific information in each study. All data were extracted by one author and checked by two other authors. The extracted data were then mapped simultaneously using the thematic analysis method for identifying patterns and finding themes in the data. Using this method, after data from various sources were obtained and analysed, the data were then arranged in a structured and systematic manner into a discussion that addressed the research questions. The data were presented in a structured and systematic tabular form.

**Figure 1.**
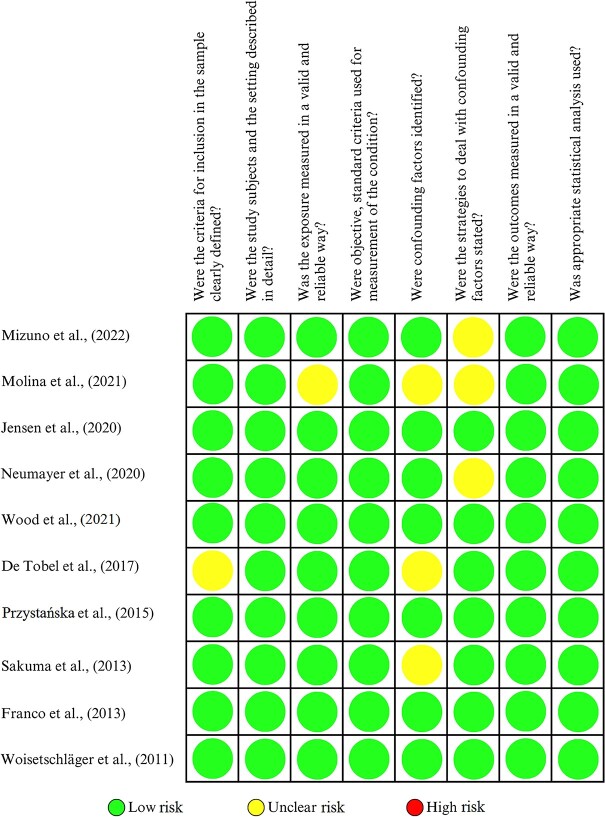
Critical assessment using the Joanna Briggs Institute checklist for analytical cross-sectional studies.

**Figure 2.**
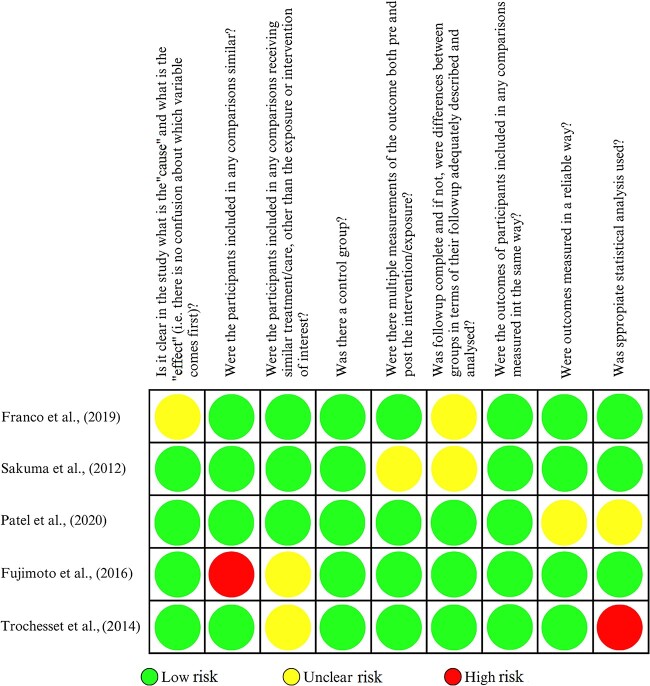
Critical assessment using the Joanna Briggs Institute checklist for quasi-experimental studies.

**Figure 3.**
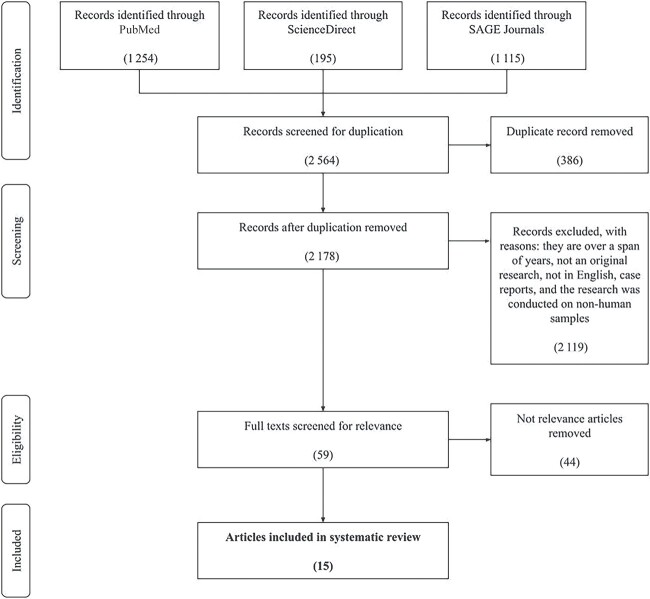
PRISMA flow chart.

### Quality assessment

Critical appraisal is a systematic assessment of research in terms of its validity, results, and relevance. The included articles were assessed using the Joanna Briggs Institute (JBI) critical assessment tool. The JBI checklist for transversal/cross-sectional studies (Figure 1) consists of eight questions, and that for quasi-experimental studies (Figure 2) consists of nine questions. Potential responses include “yes”, “no”, “unclear”, and “not applicable”. Each criterion with a “yes” response is given 1 point, and other responses are not assigned a point. If the number of “yes” responses is above 50%, then the article is considered to be of high quality. Articles were categorized as low, medium, and high quality and assessed by the authors to reduce the risk of bias.

## Results

### Search results

A total of 2 564 articles were identified, with 1 254 articles in PubMed, 195 articles in ScienceDirect, and 1 115 articles in the SAGE Journals database. The initial selection was carried out by removing 386 duplicate articles. Subsequent selection by applying the inclusion and exclusion criteria resulted in a total of 59 articles, after 2 119 articles were excluded. The final selection was carried out by reading the entire contents of the article to determine the suitability of the research in relation to the title, leading to the inclusion of 15 research articles in the analysis after 44 articles were filtered out because they were irrelevant. The article selection process is shown as a flow chart in Figure 3. A total of 15 articles were then compiled and analysed sequentially from the most recent publication year.

### Article characteristics

**Figure 4.**
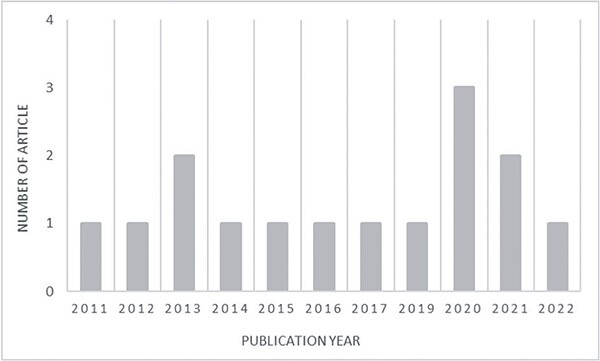
Number of articles on the use of oral and maxillofacial radiographs in virtual autopsy and forensic odontology published from 2011 to 2022.

A total of 15 articles were included in this systematic review. Table 1 shows the characteristics of the articles and summarizes the search results for the included articles. Most articles came from reputable journals Q2 with eight articles (53.33%), followed by Q1 with five articles (33.33%) and Q3 with two articles (13.33%). The designs of the included studies were as follows: 11 transverse/cross-sectional studies, and four quasi-experimental studies. The articles were mostly from Western countries; eight studies were from Europe (Spain, Denmark, Austria, Belgium, Poland, and Sweden), four were from Japan, two were from the Americas (the USA and Brazil), and one was from India. The earliest article included in this study was published in 2011. Since then, an increasing number of published articles have examined the use of virtual autopsy in forensic odontology, with at least one review being published consecutively every year from 2011 until 2022. This information is shown as a diagram in Figure 4.

The use of oral and maxillofacial radiographs in forensic odontology in the included articles can be classified into several categories: (i) identification of teeth using a CT scan [24–30]; (ii) age estimation using CT scanning [31]; (iii) tooth identification using CBCT [30, 32–35]; (iv) age estimation using CBCT [33, 36]; and (v) age estimation using MRI [37, 38].

### Risk of bias

The risk of bias assessment was conducted in 15 studies using the JBI critical appraisal checklist. Ten of these studies were assessed by an analytical cross-sectional study critical appraisal checklist, and all were found to be high-quality articles. The remaining five articles were quasi-experimental studies and were also found to be high-quality articles.

**Table 1:** Summary of articles included in this systematic review (*n* = 15).

Reference	Journal reputation	Origin	Aims	Study design	Sample		Technique	Result
					Specimen	Status		
Mizuno et al. (2022) [29]	Q1	Japan	To illustrate the oral characteristics of Japanese methamphetamine users using forensic autopsy reports and postmortem CT images.	Transversal study	CT images from 3 338 decedents at two forensic medicine departments in Japan	Living material	CT scan	• CT images provide information that allows to detect fractures, number of teeth, dental restoration, severe caries, and alveolar bone absorption, regardless of mortality status. • CT provide information to assess the periodontal status.
Molina et al. (2021) [36]	Q1	Spain	To use CBCT images in calculating the pulp/tooth volume ratio in a Spanish population to increase the applicability of this imaging technique for estimating dental age in living individuals.	Transversal study	313 teeth (incisors, canines, and lower premolars) from 107 patients: 56 women and 51 men with a mean age of 44 ± 14 years (range, 14–70 years)	Living material	CBCT	• CBCT takes less time and achieves higher inter-examiner reliability. • CBCT is an accurate tool for age estimation with a relatively lower radiation dose.
Wood et al. (2021) [30]	Q2	Canada	Describe how medical CT images were used with CBCT software in the context of a small multiple fatality incident involving a small aircraft with seven deceased individuals.	Transversal study	Four adults and three children full postmortem CT scan	Deceased	CT scan and CBCT	• CT scan can be performed very quickly and is time-saving in comparison to traditional intra-oral radiograph imaging. • This technique is beneficial to all parties, and it removes the biological hazards from the operator since the body can be imaged in the body bag. • CT images are also able to take multiple projections with a single exposure very quickly.
Jensen et al. (2020) [28]	Q2	Denmark	To mentioned where postmortem CT has advantages and disadvantages in recording dental postmortem data for identification purposes.	Transversal study	Postmortem CT files and conventional dental identification checks from 100 forensic identification cases in University of Copenhagen School of Medicine	Deceased	CT scan	• CT scan can identify the presence or absence of teeth. • CT scan can detect abutments and pontics easily. • This technique can easily detect metal, but cannot distinguish whether amalgam, gold, or metal-coloured filling. • The accuracy and sensitivity of this technique are high, especially in the case of endodontically treated teeth. • The resulting image does not appear with sharp edges or demarcation. • CT scan is difficult to detect small restorations and tooth-coloured restorations. • The presence of artefacts can make identification difficult. • CT scan is useful as an adjunct to conventional identification. • CT scan allows identification by non-invasive methods.

(*Continued*)

**Table 1:** Continued.

Reference	Journal reputation	Origin	Aims	Study design	Sample		Technique	Result
					Specimen	Status		
Patel et al. (2020) [35]	Q3	India	To highlight the relationship between forensic and endodontic science by illustrating CBCT recordings as legal evidence for forensic analysis and observing macroscopic and stereo-microscopic changes at elevated temperatures in endodontically treated teeth.	Quasi-experimental study	40 post-extraction mandibular premolars for orthodontic purposes in the Acteon population, France	Living material	CBCT	• CBCT can identify 3D changes in the root, root canal morphology, root canal filling material, and anatomical variations such as root canal loss. • CBCT can detect 3D changes caused by thermal stress in endodontically treated teeth. • Identification using CBCT is a non-invasive method.
Neumayer et al. (2020) [38]	Q1	Austria	To investigate the reliability of multi-factorial age estimation based on MRI data of hand, wisdom teeth, and clavicle bone with a reduced acquisition time.	Transversal study	MRI data from 34 Caucasian male volunteers with an age range of 13.37 to 24.05 years	Living material	MRI	It is possible to reduce the total MRI acquisition time between 4 and 5 min while allowing reliable age estimation.
Franco et al. (2019) [34]	Q1	Brazil	To validate the prevalence of INTERPOL-coded dental identifiers in CBCT and panoramic scan samples. Measure the prevalence correspondence of INTERPOL-coded dental identifiers between CBCT and panoramic scans. Measure in the study sample the relationship between the detected INTERPOL-coded tooth identifier and the position of the respective tooth.	Quasi-experimental study	100 CBCT and 100 panoramic scans were taken on the same day from 100 Brazilian subjects (35 men and 65 women) aged 18 to 65 years	Living material	CBCT	The most frequently detected INTERPOL codes by CBCT are the presence/absence of teeth, fixed orthodontic appliances, and tooth-coloured fillings.
De Tobel et al. (2017) [37]	Q2	Belgium	To develop specific MRI staging techniques for the development of third molars. To evaluate the age estimation performance of the Bayesian method using MRI.	Transversal study	309 Belgian and Dutch volunteers (163 women, 146 men)	Living material	MRI	The staging method on MRI shows reproducibility and performance comparable with the CT scan method.
Fujimoto et al. (2016) [27]	Q3	Japan	To develop a new method of victim identification aimed at preparing a DVI system and to assist in the search for missing persons.	Quasi-experimental study	100 volunteer patients (50 females and 50 males) from Fujimoto Clinic for Oral and Maxillofacial Surgery	Living material	CT scan	Large metal prostheses will create streak artefacts on CT images, which may hinder the reconstruction process.
Przystańska et al. (2015) [33]	Q2	Poland	To assess tooth status, tooth morphology, and dental pathology as well as tooth wear and enamel hypoplasia based on visual examination and stereomicroscopic examination. To estimate the dental age at death.	Transversal study	13 graves with skeletal remains of 15 individuals (8 adults: 3 males and 5 females, and 7 children) from the Strzyzow Culture burial site in Rogalin	Deceased	CBCT	• CBCT can detect large carious lesions extending into the pulp cavity and root surface. • CBCT can show growth and eruption of deciduous teeth. • CBCT provides high-quality images with a non-destructive method for age estimation.

(*Continued*)

**Table 1:** Continued.

Reference	Journal reputation	Origin	Aims	Study design	Sample		Technique	Result
					Specimen	Status		
Trochesset et al. (2014) [32]	Q2	USA	To demonstrate a protocol for generating images from reformatted CBCT volumes of jaw specimens obtained from corpses previously used in anatomical training and showing reformatted images similar to conventional or digital periapical or bitewing radiograph images taken on the same cadaver specimen.	Quasi-experimental study	Digital image of periapical and bitewing teeth of half head and mandible of cadaver	Deceased	CBCT	• The resulting image can be adapted to the anatomical area, the field of view, and angular orientation of the comparison (i.e. antemortem) image, eliminating distracting features. • This protocol requires scan times of under 30 s, fewer image artefacts, low image reconstruction times, unit mobility, and significantly lower equipment costs. • CBCT is a time-saving and resource-saving technique so it can be used in situations of natural disasters or teeth that cannot be scanned with conventional techniques.
Sakuma et al. (2013) [31]	Q2	Japan	To evaluate whether reconstructed MDCT images obtained before a forensic autopsy are useful for estimating age at death.	Transversal study	CT images of 136 bodies with the known age of death (range, 14–79 years)	Living material	MDCT	• MDCT can identify the boundary between the pulp chamber and dentin thereby eliminating errors. • Age estimation using MDCT can reduce identification time. • This technique is not suitable for use on teeth prone to caries because the metal restorative material used can cause interference. • The reconstructed MDCT images have the potential to be used in additional methods when combined with other investigations.
Franco et al. (2013) [26]	Q2	Belgium	To verify whether a virtual autopsy based on a full-body CT scan of the postmortem can be used to perform dental charting of postmortem using the INTERPOL code.	Transversal study	103 full body CT scan in the Department of Radiology before a forensic autopsy by the Department of Forensic Medicine KU Leuven University Hospital, Belgium	Deceased	CT scan	• CT scan is difficult to differentiate various restorative materials. • CT scan can detect dental information such as the presence/absence of teeth, malposition, crowding, and diastema. • 3D image reconstruction allows for the best observations. • CT scan can also detect abnormalities such as crown fracture, root remnant, attrition, and caries. • CT scan can detect other information such as tongue and lip piercings, surgical plates, mandibular torus, periapical abnormalities, and severe periodontal bone loss. • CT scan can be used as an adjunct in human dental identification procedures.

(*Continued*)

**Table 1:** Continued.

Reference	Journal reputation	Origin	Aims	Study design	Sample		Technique	Result
					Specimen	Status		
Sakuma et al. (2012) [25]	Q2	Japan	To differentiate between enamel and resin composites with HU differences shown on 16 sections of MDCT images taken from unidentified bodies.	Quasi-experimental study	15 extracted human teeth (2 incisors, 2 premolars, and 11 molars)	Living material	MDCT	• The composite resin is visible, and the reconstructed CT image helps to delineate the area of the composite resin on the dental chart. • CT scan is prone to artefacts and metal prostheses can hinder accurate analysis. • Reconstructed CT images can contribute to tooth identification, especially in cases where it is difficult to detect composite resin on external examination. • CT scan can be used as an additional method of dental identification when combined with an external examination.
Woisetschläger et al. (2011) [24]	Q1	Sweden	To evaluate the use of ultra high-resolution CT to radiologically determine tooth morphology and filler properties after exposure to high temperatures in units of HU.	Transversal study	122 previously prepared and restored molars with 10 different filling materials (temporary fillings, permanent fillings, liners, and adhesives)	Living material	CT scan	• CT scan can visualize restorative material even if the tooth is fractured or dislocated. • Identification using CT scan is a non-invasive method because it can be done without damaging any part of the body. • Amalgam produces streak and blooming artefacts in images. • Ultra-high-resolution CT can improve the identification process of fire victims.

CT: computed tomography; CBCT: cone beam CT; MRI: magnetic resonance imaging; DVI: disaster victim identification; MDCT: multidetector CT; HU: Hounsfield unit.

### Dental identification from CT scanning

CT scanning is considered to be an effective method for describing tissue remains, personal belongings, prostheses, and general dental information on corpses [23]. Woisetschläger et al. [24] reported that dental identification using high-resolution CT (HRCT) can improve documentation for identification purposes in fire victims. Sakuma et al. [25] reported that identification using MDCT can help to clearly describe an area of composite resin, so can contribute to dental identification, especially in cases where it is difficult to detect composite resin using external examination. A study conducted by Franco et al. [26] indicated that CT scanning can detect dental information, such as the presence/absence of teeth, dental pathology, prostheses, and other information such as lip and tongue piercings, as well as abnormal lesions such as the mandibular torus. The study also mentioned that CT scanning methods had difficulty distinguishing between different types of restorative materials. This finding is in accord with another study conducted by Jensen et al. [28], which indicated that the use of CT scanning in dental identification has high accuracy and sensitivity, particularly in endodontic treatment cases. Wood et al. [30] reported that CT is a time-saving alternative in comparison to traditional intra-oral radiograph imaging. A study by Mizuno et al. [29] indicated that CT images can detect fractures, number of teeth, dental restoration, severe caries, and alveolar bone absorption, regardless of mortality status. Two previous studies reported that, in dental identification using CT scanning, the use of metal restorations can create artefacts in the image that may inhibit the reconstruction process [27, 28].

### Age estimation from CT scanning

A study conducted by Sakuma et al. [31] revealed that MDCT can easily distinguish the pulp cavity from the hard tissue of the tooth so that it can be used in age estimation. The study also mentioned that this technique is not suitable for use on teeth that are prone to caries and have been restored with metal, because this can cause interference with the identification process.

### Dental identification from CBCT

Two articles reported that CBCT is capable of detecting normal and abnormal variations such as the presence/absence of teeth, tooth characteristics, as well as anatomical variations such as the loss of root canals, and pathological abnormalities such as large carious lesions that extend into the pulp cavity or root surface [34, 35]. Franco et al. [34] reported that CBCT can detect some dental information, such as the presence or absence of teeth, fixed orthodontic appliances, tooth-coloured fillings, and metal restorations. Patel et al. [35] reported that CBCT can provide information about root and root canal morphology.

### Age estimation from CBCT

Studies on the use of CBCT in age estimation using dental data have been identified. Przystańska et al. [33] reported that CBCT can be used to identify tooth age at death. In another study, Molina et al. [36] reported that CBCT is an accurate imaging technique for measuring tooth volume with a relatively low radiation dose. The study also indicated that the use of CBCT in age estimation requires less time and had a higher level of reliability compared with conventional methods [36].

### Age estimation from MRI

All age estimations performed in the articles included in this review indicated that the mineralization stage of the third molar can be identified using MRI data. De Tobel et al. [37] reported that age estimation based on a proposed staging method for third molars on MRI exhibited comparable reproducibility and performance to CT scan. A study of age estimation with MRI was conducted by Neumayer et al. [38], and indicated that age estimation using MRI can be performed only with a total acquisition time of between 4 and 5 minutes.

## Discussion

The process of identifying unknown remains in the field of forensics must follow the best practice standards and should always include the collection of all postmortem data. The dental autopsy process requires comparison with the antemortem data of the individual and final reconciliation of the data collected. In this case, a complete dental autopsy and peer-reviewed evaluation are essential for achieving individual identification. In the current review, most of the articles discussing the use of oral and maxillofacial radiographs as virtual autopsy in forensic odontology reported that 3D methods provide a valuable non-invasive tool in the process of identifying individuals using dental data [24, 26, 28, 31, 33, 35, 36]. Fujimoto et al. [27] reported that CT scanning is a reliable, fast, and easy-to-apply tool for the identification of victims and missing persons. Wood et al. [30] mentioned that this technique can remove the contamination risk of biological hazards from the corpses to the operator because the body can be imaged in the body bag. Neumayer et al. [38] reported that MRI is a reliable method for age estimation. This is in line with a study using CBCT conducted by Molina et al. [36], which reported that CBCT is an accurate tool for age estimation. However, this contrasts with a study by Jensen et al. [28], which mentioned that the use of oral and maxillofacial radiography for virtual autopsy can currently only be used as an additional tool in dental data collection and cannot yet stand alone as an identification method. The studies included in the current review indicate that 3D oral and maxillofacial radiography methods, such as CT scanning, MRI, and CBCT, provide reliable and accurate tools for individual identification in forensic odontology, but that these techniques cannot yet be applied as the main method in the process of a forensic autopsy.

The current review identified several articles describing the use of CT scanning in forensic odontology. CT scanning is reported to be able to identify dental information such as the presence/absence of teeth, dental abnormalities such as malposition, crowding, and diastema, as well as personal information such as lip and tongue piercing [26]. In addition, Mizuno et al. [29] reported that CT images provide information that allows odontologists to detect fractures, dental restoration, severe caries, and periodontal status. CT scanning is also used for dental age estimation [31]. Another study by Woisetschläger et al. [24] mentioned that dental identification using HRCT can visualize restorative materials well, even if the tooth is fractured. A study by Franco et al. [26] yielded different findings, indicating that differences in the composition of dental restoration materials were difficult to distinguish in CT images. This discrepancy in findings may have occurred because HRCT produces thinner slices than CT scanning, which enables it to produce more detailed images. Furthermore, this contradictory result may have occurred because the study conducted by Woisetschläger et al. [24] was performed on extracted teeth under optimal conditions for distinguishing minor details that are difficult to identify in corpses in real autopsy conditions. In another study, Jensen et al. [28] reported that postmortem CT can easily detect the presence of metal-based restorations. However, the study also mentioned that the resulting postmortem CT images did not appear to have sharp edges and were not able to distinguish whether the metal restoration involved amalgam, gold, or metal-coloured fillings [28]. Difficulty in detecting the type of metal restoration may result from the presence of artefacts caused by the metal itself. Artefacts generated in images can hinder the reconstruction and identification process [26–28]. This finding is supported by Ruder et al. [39], who mentioned that the main limitation in the use of CT scanning is the emergence of artefacts from restorative materials with high radiological opacity, such as gold or amalgam. Artefacts in the CT image occur because the CT scan attenuation data are distorted by the high density of metal objects, causing inconsistencies that prevent adequate projection data calculations and eventually result in starburst artefacts [40]. However, a different finding was reported by Diehn et al. [41], who mentioned that artefacts caused by metal restorations can be reduced using the iterative metal artefact reduction reconstruction technique. In addition, this artefact reduction technique can improve the visualization of soft tissue anatomy in the oral cavity and oropharynx. To date, the use of CT scanning as a virtual autopsy method in forensic odontology activities has faced difficulty when dealing with metal-based restorations and produces indistinct edges. However, CT scanning can still reveal useful dental information, such as the presence/absence of teeth, the restorative materials used, dental abnormalities, and personal information such as lip and tongue piercings.

In the current study, we identified several articles that used the CBCT technique for individual identification, particularly in the collection of dental data. Two articles mentioned that CBCT is also capable of detecting normal and abnormal variations, such as the presence/absence of teeth, and tooth characteristics, as well as anatomical variations such as loss of root canals and pathological abnormalities, such as large carious lesions that extend into the pulp cavity or root surface [33, 35]. Patel et al. [35] reported that this non-invasive imaging technique is helpful for gaining an accurate understanding of 3D changes caused by thermal stress in endodontically treated teeth. The study also mentioned that CBCT can identify 3D changes in roots, root canal morphology, root canal filling material, and anatomical variations such as root canal loss [35], because CBCT makes it possible to view 3D anatomical features by reconstructing projection data to provide interrelationship images in three orthogonal planes (axial, sagittal, and coronal) [42]. Franco et al. [34] reported that CBCT can also detect other types of dental information such as fixed orthodontic appliances, tooth-coloured fillings, and metal-based restorations. This finding differs from the results of a study conducted by Vega-Dominguez et al. [43], which indicated that metal restorations can produce various artefacts on CBCT images that can raise doubts in the assessment. Another study by Jain et al. [44] indicated that the lower CBCT accuracy may be caused by metal restorations or beam-hardening artefacts. Although CBCT assessment can be influenced by artefacts produced by metal-based restorations, the use of CBCT can identify dental data effectively, such as tooth characteristics, the depth of caries, and, particularly in endodontically treated teeth, both root canal morphology and root canal filling material, as well as anatomical variations such as loss of root canal. This is possible CBCT allows viewing of images in three orthogonal planes.

Regarding dental age estimation, the current review identified several studies that used CBCT to estimate age. In one study, Molina et al. [36] used the ratio between the pulp chamber and the crown of the tooth in age estimation, reporting that CBCT is an accurate imaging technique for measuring tooth volume with a relatively low radiation dose. This finding is in line with a study by Oscandar et al. [45], which reported that CBCT could be used to determine the correlation between pulp chamber volume and chronological age in a Deutero Malay population. Both of these studies were conducted in a faculty of forensics, which had adequate facilities. Dental age estimation is a particularly valuable tool for victim identification in disaster situations. However, Kumagai et al. [46] reported that the use of CBCT is difficult in areas that have limited access to electricity. The studies reviewed above indicated that CBCT can be used to identify pulp chamber volume, which is useful for dental age estimation with a relatively lower radiation dose. However, the use of this method is currently difficult in areas with limited access to electricity.

In terms of age estimation on the basis of dental data, we identified two studies in which an analysis of dental mineralization stage was performed using MRI. The age estimation method using MRI is a non-invasive alternative with no-ionizing radiation. The use of MRI in the analysis of third molar development in age estimation revealed comparable reproducibility and performance when compared with orthopantomography (OPG) [37]. This is in line with a study by Baumann et al. [47], which mentioned that MRI is a good choice for use in dental age estimation because it is better able to show the stage of molar development compared with OPG. However, a study conducted by Grabherr et al. [48] suggested that MRI is not widely used in modern forensic imaging because the maintenance and handling of the MRI unit are very expensive compared with other methods. Widek et al. [49] reported that age estimation using MRI can be hampered if metal restoration is present, which can affect the process of tooth mineralization analysis because of limited access to the entire root. Thus, MRI has been shown to be useful for analysing mineralization stages of third molars in age estimation, with good reproducibility and performance when not dealing with metal restorations. However, the use of this method requires maintenance and expensive unit handling.

The time required for clinical dental autopsy typically ranges from 1 to 4 h, depending on the complexity of the case being treated. In the current review, several articles mentioned that the use of oral and maxillofacial radiography in virtual autopsy and forensic odontology can increase the effectiveness and speed of autopsy times, especially in mass disasters. A study conducted by Troschesset et al. [32] reported that the dental identification method using CBCT requires a scan time of under 30 seconds with unit mobility and much lower equipment costs. Another study by Neumayer et al. [38] mentioned that by using MRI, the total scan acquisition time can be reduced by 4–5 minutes. Similar results were reported by Molina et al. [36], who reported that the use of CBCT in age estimation required less time with a high level of reliability compared with conventional methods. This result is in contrast to the findings of a study by Das and Jena [50], who reported that although virtual autopsy using 3D oral and maxillofacial radiography requires less “handling” time compared with conventional autopsy, the use of the method has limitations, such as difficulty detecting minor trauma lesions, infection status, and other pathological conditions. Furthermore, although 3D scanning requires less “handling” time, the “interpretation” process usually takes time and often requires the involvement of a third-party expert. However, less “handling” time in an autopsy can provide an appropriate solution for some conditions in which religious beliefs require an immediate burial. Thus, the use of oral and maxillofacial radiographs in virtual autopsy and forensic odontology may increase the speed of autopsy, particularly in disaster situations, because less “handling” time is required compared with conventional autopsy, while maintaining high reliability. In addition, 3D oral and maxillofacial radiography has been used as a valuable tool for non-invasive autopsy, with each technique having advantages and disadvantages.

The studies included in the current review indicated that the use of oral and maxillofacial radiography for virtual autopsy in forensic odontology has the advantage of being able to assist in the identification of individuals using dental data, as well as performing age estimation in a non-destructive and non-invasive way. This technique is also reported to speed up the identification handling process and has good accuracy, especially for the identification of teeth with root canal treatment. Identification using this technique also enables improved documentation. Limitations of the use of oral and maxillofacial radiography as virtual autopsy methods include the high cost of maintenance of the unit. In addition, the resulting images typically do not have clear boundaries, and some dental information can be difficult to identify in cases where metal restoration causes artefacts in the image, hindering the identification process.

In the current review, we identified more studies using CT scanning in forensic identification compared with other virtual imaging methods. This finding is in line with a study by Grabherr et al. [48], who reported that CT scanning is one of the most commonly used radiological modalities in modern forensic imaging. Although many studies have used CT scanning to perform dental autopsies, Grabherr et al. [48] also mentioned the use of MR scanners, which are less widely available than CT devices, and less commonly used in modern forensic imaging. Izam and Auerkari [51] reported that there has been an increase in the use of CBCT for postmortem examinations in recent years. However, to date, no studies have reported the distribution of access to these imaging methods. Thus, CT scanners, CBCT, and MR scanners might not be present at some forensic medicine institutes, and technology for performing virtual autopsy may be inaccessible in many parts of the world.

## Study limitations

One limitation of this systematic review is that some articles related to this study may not have been included because of the limitations of the search criteria and the year of publication. In addition, because this field of knowledge is currently developing very quickly, some of the included studies may not be relevant in the future. Most of the studies included in this review only conducted research on living material, including extracted teeth. Studies performed on either extracted teeth or living persons may be more likely to report the advantages of these methods, because of the optimal conditions for providing detailed results. Therefore, we recommend approaches that involve the application of these methods to “real-world” settings, particularly real autopsies with corpses.

## Conclusion

The studies included in the current review revealed several oral and maxillofacial radiographic techniques that have been used as fundamental methods of virtual autopsy in forensic odontology: CT scans (including MDCT), CBCT, and MRI. Several uses of oral and maxillofacial radiographs as virtual autopsy methods were identified as having advantages as well as limitations in their use in the field of forensic odontology. Most of the articles included in the review indicate that these techniques cannot yet stand alone as identification methods. Although virtual autopsy cannot yet provide a complete alternative to a conventional autopsy, this approach can provide a useful adjunct tool for gathering dental evidence.
